# Fumigant Activity of the *Psidium guajava* Var. Pomifera (Myrtaceae) Essential Oil in *Drosophila melanogaster* by Means of Oxidative Stress

**DOI:** 10.1155/2014/696785

**Published:** 2014-11-12

**Authors:** Antonio Ivanildo Pinho, Gabriel Luz Wallau, Mauro Eugenio Medina Nunes, Nadghia Figueiredo Leite, Saulo Relison Tintino, Litiele Cezar da Cruz, Francisco Assis Bezerra da Cunha, José Galberto Martins da Costa, Henrique Douglas Melo Coutinho, Thais Posser, Jeferson Luis Franco

**Affiliations:** ^1^Laboratório de Microbiologia e Biologia Molecular, Universidade Regional do Cariri, 63105-000 Crato, CE, Brazil; ^2^Centro Interdisciplinar de Pesquisas em Biotecnologia (CIPBIOTEC), Universidade Federal do Pampa, Campus São Gabriel, Avenida Av Antonio Trilha 1847, Centro, 97300-000 São Gabriel, RS, Brazil; ^3^Laboratório de Pesquisa em Produtos Naturais, Universidade Regional do Cariri, 63105-000 Crato, CE, Brazil

## Abstract

The guava fruit,* Psidium guajava* var. pomifera (Myrtaceae family), is a native plant from South America. Its leaves and fruits are widely used in popular medicine in tropical and subtropical countries.* Drosophila melanogaster* has been used as one of the main model organisms in genetic studies since the 1900s. The extensive knowledge about this species makes it one of the most suitable organisms to study many aspects of toxic compound effects. Due to the lack of studies on the effects of the bioactive compounds present in the* P. guajava* var. pomifera essential oil, we performed a phytochemical characterization by CG-MS and evaluated the toxicity induced by the essential oil in the* D. melanogaster* insect model. In order to understand the biochemical mechanisms of toxicity, changes on the Nrf2 signaling as well as hallmarks of oxidative stress response were followed in the exposed flies. Our results showed that exposure of insects to the* P. guajava* oil increased mortality and locomotor deficits in parallel with an oxidative stress response signaling. Therefore, it suggested a bioinsecticidal activity for* P. guajava* volatile compounds by means of oxidative stress. Further studies are ongoing to identify which oil compounds are responsible for such effect.

## 1. Introduction

With the continual increase in the human population worldwide, one of the most challenging situations is to provide enough food to the human population. There are two possibilities to reach such endeavor: (1) increase the agricultural area or (2) optimize the production of the already cultivated fields. Insect pests are one of the most important threats for the cultivated crops causing a serious reduction in the global production [1].

Synthetic insecticides are widely used to control insect pests. However, the chemical properties of these products make them dangerous for both humans and the environment [2]. Moreover, the plasticity of insect pests makes them prone to develop resistance to many of these compounds [3]. Searching new insecticides that offer no or low risks and that are decomposed to safe compounds after its action is needed in order to overcome these issues. Plant derived insecticides can be a suitable alternative, since vegetables species have evolved molecular mechanisms that protect them against herbivorous insects and other animal species [4]. Essential oils from plant species have been reported as acting on digestive and neurological enzymes as well as with insects tegument [5, 6]. Some authors suggested that such insecticide effect is probably due to the secondary metabolites as terpenoids and phenylpropanoids [7]. An insecticidal activity of some monoterpenes as *α*-pinene, *β*-pinene, 3-carene, limonene, myrcene, *α*-terpinene, and camphene had been demonstrated in literature [8].

*Psidium guajava* (Myrtaceae family) is a native bush species from South America known as “goiaba.” There are two more common cultivated varieties of* P. guajava*:* P. guajava* var. pomifera and* P. guajava* var. pyrifera. The* P. guajava* var. pomifera produces a fruit highly appreciated in the tropical and subtropical culinary and also is used in the popular medicine [9]. Extracts from leaves and fruits of this species presented several pharmacological properties as antispasmodic, antimicrobial and anti-inflammatory [10]. Moreover, these extracts also have been used as hypoglycemic [11]. Despite the available reports on benefits of guava to human health, little is known about its potential in biotechnological applications (e.g., fumigant activity) of guava extracts, oils, and derived compounds.

In the last decade,* Drosophila melanogaster* became a model for testing toxicity* in vivo. *It is due to the fact that this species has many homologous genes with humans and can be easily kept at the laboratory allowing many assays to be performed [12–15]. Therefore,* D. melanogaster* model can be widely used for evaluating fumigant activity screenings.

In summary, considering (i) the undesired adverse effects of synthetic means of pest control to humans and the environment, (ii) the ability of plant metabolites to induce toxicity to insects, and (iii) the lack of studies on the biotechnological potential of guava fruit derived compounds, the main goal of this work was to evaluate the biological activity of the essential oil from* Psidium guajava* var. pomifera and investigate the mechanism by which this oil promotes toxicity using the model organism* D. melanogaster*. Toxicity was evaluated as mortality and locomotor deficits. In parallel, oxidative stress signaling markers were determined in order to search for potential mechanisms of toxicity induced by the essential oil in* Drosophila*.

## 2. Materials and Methods

### 2.1. Plant Material

The plant material of* Psidium guajava* var. pomifera, was collected in the Horto Botânico de Plantas Medicinais do Laboratório de Pesquisa de Produtos Naturais (LPPN) of Universidade Regional do Cariri (URCA), Ceará, Brazil. The plant material was identified, and a voucher specimen was deposited in the Herbarium Dardano Andrade Lima of URCA, under number 3930.

### 2.2. Collection of Essential Oil

Leaves of* Psidium guajava* var. pomifera L. were collected, chopped into pieces of approximately 1 cm^2^, and placed in a 5-liter glass flask. The leaves were extracted with a clevenger apparatus, according to the method described by de Matos [16], giving a yield of 0.05%.

### 2.3. GC—MS Analysis

Oil analysis was performed using a Shimadzu GC MS—QP2010 series (GC/MS system): Rtx-5MS capillary column (30 m × 0.25 mm, 0.25 *μ*m film thickness); helium carrier gas at 1.5 mL/min; injector temperature 250°C; detector temperature 290°C; column temperature 60–180°C at 5°C/min, and then 180–280°C at 10°C/min (10 min). Scanning speed was 0.5 scan/s from* m/z* 40 to 350; split ratio (1 : 200); injected volume: 1 *μ*L of 25 *μ*L essential oil/5 mL CHCl_3_ (1 : 200); solvent cut time = 2.5 min. The mass spectrometer was operated using 70 eV ionization energy. Identification of individual components was based on their mass spectral fragmentation based on mass spectral library NIST 08, retention indices, and comparison with published data.

### 2.4. *Drosophila* Stock and Culture

*D. melanogaster* (Harwich strain) was obtained from the National Species Stock Center, Bowling Green, OH. Flies were reared in 2.5 × 6.5 cm^2^ glass bottles containing 10 mL of standard medium (1% w/v brewer's yeast; 2% w/v sucrose; 1% w/v powdered milk; 1% w/v agar; 0.08% v/w nepagin) at constant temperature and humidity (25 ± 1°C; 60% relative humidity, resp.). All experiments were performed with the same strain.

### 2.5. Essential Oil Exposure and Flies Survival Assay

The exposure of flies to the essential oil was performed by a fumigation protocol as described: adult flies (males and females) were placed in 330 cm^3^ glass vials, containing a filter paper soaked with 1% sucrose in distilled water at the bottom. A counter-lid of polyethylene terephthalate (PET) was introduced on the screw cap of the vial, to which a filter paper was fixed at the inner side of the cap for application of different doses of essential oil. By doing this, the flies feed and hydrate on sucrose solution at the bottom of the vials and the essential oil is allowed to volatilize from the top in order to reach flies' respiratory system. The vials received the following treatments: 1% sucrose (control) and 3, 7.5, 15, 23.5, and 30 *μ*g/mL of essential oil. The final concentration of the essential oil was estimated by approximation, taking into account the volume (in microliters) of the oil applied to a glass vial with a final volume equivalent to 330 mL. Readings of flies' survivorship were taken at 6, 12, 24, and 48 h. Results are presented as percentage (%) of live flies (mean ± SD) obtained from three independent experiments.

### 2.6. Locomotor Assay

The locomotor capacity was evaluated by following the negative geotaxis behavior as described by Coulom and Birman [17] with some modifications. Twenty adult flies (1–4-day old; both genders) were subjected to essential oil exposure as detailed above. After treatments were finished, flies were immobilized on ice for 1-2 minutes and placed separately in vertical glass columns (length, 25 cm; diameter, 1.5 cm). After 30 min recovery, flies were gently tapped to the bottom of the column and the number of flies that reached 6 cm of the column (top) and flies that remained below this mark (bottom) were registered. The assays were repeated three times for each fly. Results are presented as number of flies on top (mean ± SD) obtained from three independent experiments.

### 2.7. Oxidative Stress Markers

Oxidative stress was determined by measuring lipid peroxidation, reactive oxygen species formation (ROS), nonprotein thiols (NPSH), and protein thiols (PSH). Byproducts of lipid peroxidation were quantified by the thiobarbituric acid reactive substances method (TBARS) following Ohkawa et al. [18] with few modifications. Briefly, 20 flies from each treatment were homogenized in 1 mL of phosphate buffer 0.1 M pH 7.0 and centrifuged at 1000 g during 5 min at 4°C. Immediately after centrifugation, the supernatant was incubated in acetic acid 0.45 M/HCl buffer pH 3.4, containing thiobarbituric acid 0.28%, SDS 1.2%, at 95°C during 60 min for color development, and then absorbance was measured at 532 nm. Malondialdehyde (0–3 nmol) was used as standard. The 2,7-dichlorofluorescein diacetate (DCFDA) oxidation was used as a general index of ROS formation following Pérez-Severiano et al. [19]. The fluorescence emission of DCF resulting from DCFDA oxidation was monitored at an excitation wave length of 485 nm and an emission wavelength of 530 nm in a multimode plate reader (EnsPire PerkinElmer, USA). Protein and nonprotein thiols were determined according to the method described by Ellman et al. [20] and adapted to our lab conditions. In summary, after treatments were finished, flies were homogenized in 0.5 M perchloric acid and centrifuged at 5000 g for 5 min at 4°C. The NPSH content was determined in the supernatant while the pellet was used for PSH measurement. Total protein was quantified according to Bradford [21].

### 2.8. Enzymatic Assays

For antioxidant enzymes activity, groups of 20 flies were homogenized in 1 mL 0.1 M phosphate buffer, pH 7.0, and centrifuged at 20.000 g for 30 min. The resulted supernatant was used for determination of glutathione S-transferase (GST), catalase (CAT), and superoxide dismutase (SOD) according to methods described earlier [22]. Glutathione S-transferase (GST; EC 2.5.1.18) activity was assayed following the procedure of Habig and Jakoby [23] using 1-chloro-2,4-dinitrobenzene (CDNB) as substrate. The assay is based on the formation of the conjugated complex of CDNB and GSH at 340 nm. The reaction was conducted in a mix consisting of 100 mM phosphate buffer pH 7.0, 1 mM EDTA, 1 mM GSH, and 2.5 mM CDNB. Catalase (CAT; EC 1.11.1.6) activity was assayed following the clearance of H_2_O_2_ at 240 nm in reaction media containing 50 mM phosphate buffer pH 7.0, 0.5 mM EDTA, 10 mM H_2_O_2_, and 0.012% TRITON X100 according to the procedure of Aebi [24]. Superoxide dismutase (SOD, EC 1.15.1.1) activity was assayed following the procedure of Kostyuk and Potapovich [25]. The assay consists in the inhibition of superoxide-driven oxidation of quercetin by SOD at 406 nm. The complete reaction system consisted of 25 mM phosphate buffer, pH 10, 0.25 mM EDTA, 0.8 mM TEMED, and 0.05 mM quercetin. All enzyme activities were performed at room temperature (25 ± 1°C) using a Thermo Scientific Evolution 60s UV-vis spectrophotometer. Total protein was quantified according to Bradford [21].

### 2.9. Western Blot Analysis of Nrf2/NQO-1/HSP70 Signaling Pathway

Protein expression was determined by Western blotting according to Posser [26] with minor modifications. Thirty flies were homogenized at 4°C in 300 *μ*L of buffer (pH 7.0) containing 50 mM Tris, 1 mM EDTA, 0.1 mM phenylmethylsulfonyl fluoride, 20 mM Na_3_VO_4_, 100 mM sodium fluoride and phosphatase inhibitor cocktail (Sigma, MO). The homogenates were centrifuged at 1000 g for 10 min at 4°C and the supernatants (S1) collected. After protein determination (following Bradford [21]) using bovine serum albumin as standard, *β*-mercaptoethanol and glycerol were added to samples to a final concentration of 8 and 25%, respectively, and the samples were frozen until further analysis. Proteins were separated using SDS-PAGE with 10% gels and then electrotransferred to nitrocellulose membranes as previously described by Posser [26]. Membranes were washed in Tris-buffered saline with Tween (TBST; 100 mM Tris-HCl, 0.9% NaCl, and 0.1% Tween-20, pH 7.5) and incubated overnight (4°C) with different primary antibodies (Santa Cruz Biotechnology, TX), all produced in rabbit (anti-Nrf2, anti-NQO-1, anti-HSP70 anti-*β*-actin; 1 : 1000 dilution in TBST). Following incubation, membranes were washed in TBST and incubated for 1 h at 25°C with HRP-linked anti-rabbit-IgG secondary specific antibodies (Sigma, MO). The immunoblots were visualized in the Image Station 4000MM PRO using ECL reagent (Santa Cruz Biotechnology, TX). Immunoreactive bands were quantified using the Scion Image software and expressed as a fold change of the mean relative to control group (treated only with sucrose).

### 2.10. Statistical Analysis

Statistical analysis was performed using one-way ANOVA followed by Dunnett's* post hoc* test when necessary. Differences were considered statistically significant when *P* < 0.05. LC50 values were determined by the Trimmed Spearman-KArber method (v 1.5).

## 3. Results

### 3.1. Chemical Composition

The five most abundant compounds in the* P. guajava* essential oil are epiglobulol (19.20%), 1.8-cineole (13.31%), isoaromadendrene oxide (11.13%), caryophyllene alcohol (10.21%), and (E)-caryophyllene (9.51%), as demonstrated by the GC-MS analysis (Table 1).

### 3.2. Toxicity in* D. melanogaster*

**Figure 1:**
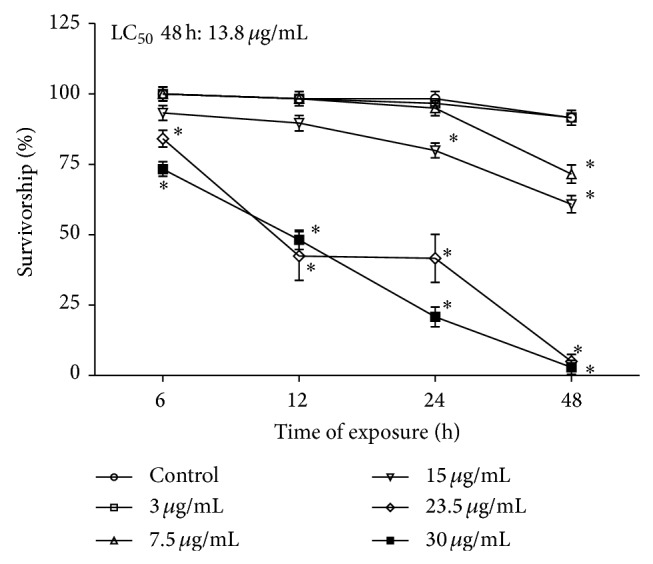
Effect of the* P. guajava *var. pomifera essential oil in the survivorship of* D. melanogaster. *Flies were exposed according to described in Section 2. Results are expressed as mean ± SD of the percentage (%) of live flies after each exposure time. ^*^ *P* < 0.05 compared to control.

**Figure 2:**
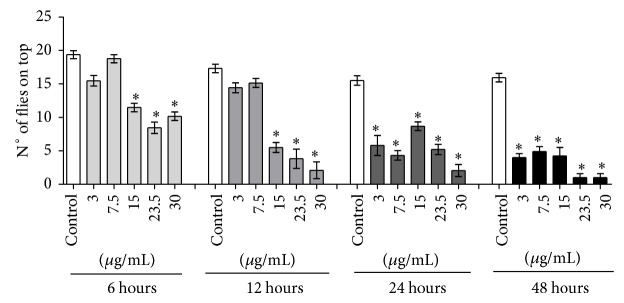
Effect of* P*.* guajava* var. pomifera essential oil in the locomotor activity (negative geotaxis behavior) of* D. melanogaster*. After treatments were finished, flies were submitted to negative geotaxis behavior test as described in Section 2. *Y*-axis represents the number of flies able to climb at least 6 cm of a glass column after 5 seconds (number of flies on top). The less the number of flies able to reach the 6 cm mark, the more affected the locomotor ability. Results are expressed as mean ± SD. ^*^ *P* < 0.05 compared to control at each exposure time.

The exposure of fruit flies to* P. guajava* essential oil by fumigation caused a significant increase in mortality. Such an effect was dependent on time and oil concentration. The calculated LC_50_ at 48 h was 13.8 *μ*g/mL (Figure 1). The concentrations of 23.5 and 30 *μ*g/mL had the most evident biocide effect, a result that could be compared with a food deprivation treatment (water only; data not shown). The highest concentrations tested killed almost the totality of flies at 48 h, showing a potent insecticide action for the essential oil. In Figure 2 the results from the locomotor activity tests are depicted. In agreement with the mortality results, a significant decrease in locomotor activity of* D. melanogaster* in the first 6 hs of treatment at 15, 23.5, and 30 *μ*g/mL can be observed. Moreover, at 48 h of exposure, the highest concentrations tested caused almost completely loss of motor ability in flies (Figure 2).

### 3.3. Oxidative Stress Markers and Antioxidant Response

**Figure 3:**
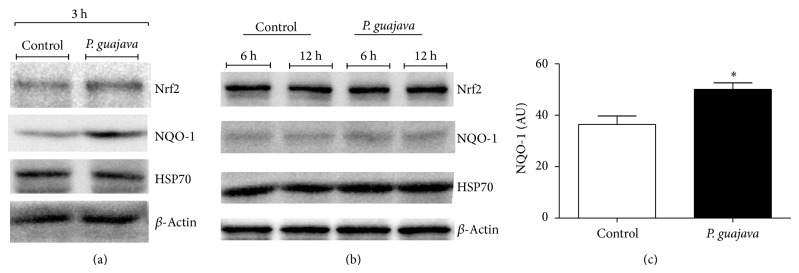
Analysis of the Nrf2/NQO-1/HSP70 signaling pathway in* D. melanogaster* exposed to* P*.* guajava* var. pomifera essential oil. After treatments were finished, samples were collected at each time point indicated and processed for western blot evaluation of each protein target. (a) Representative immunoblots for protein targets at 3 h of exposure to the essential oil. (b) Representative immunoblots for protein targets at 6 h and 12 h of exposure to the essential oil. (c) Optical densitometry of immunoreactive bands of NQO-1. Results are expressed as arbitrary units (mean ± SD). ^*^ *P* < 0.05 compared to control.

In order to clarify potential mechanisms by which* D. melanogaster* is affected by the* P. guajava* essential oil, flies were exposed to 15 *μ*g/mL of oil during 3, 6, and 12 h. Then, oxidative stress markers and the activity of antioxidant enzymes were determined (Table 2). This concentration is below the LC_50_ 48 h for* D. melanogaster*. It was possible to observe a significant increase in ROS formation at 3 h exposure to the essential oil, a result that was maintained after 6 and 12 h as well. Our results showed an increased level of TBARS after 12 h of exposure indicating that lipid peroxidation took place. The levels of protein thiols (PSH) were not changed, but nonprotein thiols (NPSH) significantly increased after 3 h of exposure, returning to basal levels at 6 and 12 h. We also evaluated the activity of three enzymes involved in the antioxidant metabolic route: GST, SOD, and CAT, as well as the expression of protein targets involved in stress response and antioxidant signaling (Nrf2, NQO-1 and HSP70). A significant increase in the activity of GST and CAT was observed when compared to control at 6 and 12 h (Table 2). However, the activity of SOD was not significantly different from the control at the time periods analyzed. As demonstrated in Figure 3, flies exposed to the essential oil presented a significant increase in the expression of NQO-1 at 3 h of exposure, indicating an early activation of the Nrf2-ARE signaling pathway. The protein levels of Nrf2 and HSP70 were not changed at the analyzed time points.

## 4. Discussion

Chemical pesticides used for insect control may be dangerous to humans and wild life. In addition, these compounds may induce insect resistance and other adverse effects, which have motivated the search for alternative forms of control [3]. In the present study we demonstrate the toxicity induced by the* Psidium guajava* var. pomifera essential oil in* Drosophila melanogaster*. The exposure of flies by the fumigation method induced substantial decreases in survivorship as well as locomotor activity. As a mechanism for the observed toxicity, the results suggest the establishment of a prooxidant condition after flies were in contact with oil derived volatile compounds. Such an effect is confirmed by increased production of reactive species and accumulation of lipid peroxidation byproducts. In addition, a clear adaptive response to oxidative stress was apparent in the oil exposed flies, since it was possible to observe an activation of antioxidant signaling pathways and increased activity of key cellular antioxidant enzymes.

Plant derived compounds are reported to induce toxicity to a wide range of insects and may interfere directly with all developmental stages of fruit fly,* Drosophila melanogaster,* and cockroaches [27, 28]. Compounds such as terpenes, flavonoids, alkaloids, steroids, and saponins are important phytochemicals when considering the insecticide activity of plant extracts [29]. In addition to acute toxicity and mortality, terpenoids and flavonoids have been also studied for their insect repellent activity [29, 30]. There are a variety of chemical compounds present in the* P. guajava *essential oil as *α*-terpineol, *α*-humulene, *β*-caryophyllene and *β*-guaiene, 1,8-cineole, caryophyllene oxide, *β*-bisabolene, aromadendrene, p-selinene, *α*-pinene, among others [31–34]. Leal et al. [35] showed the insecticidal activity of the 1,8-cineole compound obtained from the* S. aromaticum, H. martiusii, *and* Lippia sidoides *essential oils. Some authors suggested that most of the monoterpenes are nontoxic for mammals and can be considered an alternative to synthetic insecticides [36, 37]. We observed, in this study, that the* P. guajava *oil presented mono and sesquiterpenoid compounds, with the 1,8-cineole being the second most abundant (Table 1). Although we did not perform essays to evaluate the insecticide activity of each compound, the presence and abundance of the 1,8-cineole suggest that it may be one of the compounds responsible for such effect. Studies are ongoing in order to clarify the role of the different compounds presented in the essential oil tested here.

According to Ennan et al. [7] some compounds from essential oils as terpenoids and phenylpropanoids can alter the insect neurotransmitters system, including the dopaminergic and cholinergic apparatus [38, 39]. We observed a significant change in the negative geotaxis behavior of flies treated with* P. guajava *oil, which reflects in a locomotor deficit. Although we were not able to directly evaluate changes in the dopaminergic and cholinergic systems in our experimental design, some of the effects observed may be linked to a potential interaction between oil components and flies neurotransmitters pathway. In this context, it has been shown that many terpenes are known as inhibitors of the acetylcholinesterase (AchE) [39]. As reported by the same authors the *α*-terpinene found in* Salvia leriifolia *showed an AChE inhibitor effect. In general, it suggests that the terpenoid compounds found in* P. guajava *may be involved in the fumigant effect and in the damage to the locomotor apparatus.

In parallel with the induced mortality and locomotor deficits, flies exposed to* P. guajava* also showed signs of oxidative stress, including ROS and TBARS formation as well as changes in important antioxidant response systems. The cellular response to oxidative stress is mostly regulated by the Nrf2 nuclear transcription factor [40]. ROS/xenobiotics induced alterations in the cellular redox state constitute an important signal to promote adaptive responses mediated by Nrf2 [41, 42]. The upregulation of detoxifying enzymes by natural compounds appears to be related to activation of Nrf2-ARE pathway [41, 42]. The Nrf2 nuclear translocation and subsequent binding to the DNA sequence known as the “antioxidant response element, ARE” may be triggered by dissociation from the inhibitory protein Keap1 as well as by phosphorylation of serine residues at the Nrf2 protein by upstream kinases such as PKC and MAPK [42]. Among proteins that are usually involved in response to oxidative stress-driven Nrf2 activation, the NAD(P)H dehydrogenase, quinone 1 oxidoreductase (NQO-1), glutamate cysteine ligase (GCL), GST, and CAT play central role [43]. Our results showed a time dependent activation of key factors on the regulation of an antioxidant response. Since a high mortality rate at almost all doses of essential oil was apparent at the first 24 h of exposure, we measured oxidative stress markers up to 12 h, in order to have a profile of the antioxidant response in animals under* P. guajava* oil treatments. Apparently, in response to the toxicity induced by oil compounds, flies presented increased ROS levels and a peak of GSH and NQO-1 (Table 2) at the first 3 h of treatment, a phenomenon that is consistent with an early activation of the Nrf2-ARE pathway [44]. While ROS continued to increase from 3 h up to 12 h, lipid peroxidation took place only at 12 h time point (Table 2). The antioxidant enzymes GST and CAT were increased during the period of 6 h up to 12 h after essential oil treatments. Despite the increased activity of antioxidant enzymes from 6 to 12 h after administration of* P. guajava* oil, such effect did not protect flies against the late onset of lipid oxidative damage. These results clearly suggest a two-phase adaptive response to oxidative stress induced by* P. guajava* oil derived compounds. An early phase triggered by ROS induction, resulting in activation of the master regulator of cellular antioxidant response, the Nrf2 transcription factor, and a late phase, characterized by oxidative damage and increased ROS/xenobiotic detoxifying enzymes (CAT and GST). Later on, mortality and locomotor deficits accomplished the toxicity induced by the essential oil.

Glutathione S-transferase is an important antioxidant enzyme involved in phase II detoxification systems [45]. GSTs belong to a family of multifunctional enzymes that catalyze the conjugation of GSH to various other molecules and play a role in mechanisms of intracellular detoxification of endo- and xenobiotic compounds [46, 47]. The observed increase of GST activity in* Drosophila melanogaster* exposed to* P. guajava *oil can be related to an adaptive response related to enhanced elimination of toxic plant derivatives [48, 49]. Singh et al. [50] demonstrated that natural compounds are able to increase the expression of GST that together with endogenous GSH favors the elimination of plant metabolites from organisms. Catalysis plays a crucial role in the clearance of hydrogen peroxide from cells as well as for oxidative stress defense [24]. Our results demonstrated a significant increase in CAT activity in flies treated with guava essential oil (Table 2). This effect was in parallel with a rise in ROS production. The method used in the present study to detect ROS was based on the oxidation of the fluorescent dye DCFDA, which is considered a general reactive species indicator; however, hydrogen peroxide is one major species detected by this probe [51]. The observed rise in GST and CAT activity by* P. guajava* in fruit flies may be explained by a potential activation of the Nrf2 signaling pathway. In fact, an early activation of this signaling pathway was noted in flies exposed to the essential oil, by means of increased NQO-1 expression as well as a rise in GSH (Figure 3 and Table 2).

## 5. Conclusion

According to our results, the essential oil of* P. guajava *var. pomifera showed a fumigant action by compromising survivorship and locomotor activity of* D. melanogaster*. As a potential molecular mechanism of toxicity, oxidative stress appeared to be central, since markers of oxidative damage of biomolecules and a clear adaptive antioxidant response were observed in exposed flies. Therefore, our results point out to the potential application of* P. guajava* essential oil and/or its compounds as an alternative to the synthetic insecticides in agricultural and pest control practices. Additional experiments are necessary to clarify the exact mechanisms of toxicity induced by* P. guajava* oil in insects and to identify candidate compounds derived from this oil.

## Figures and Tables

**Table 1:** Chemical composition (%) of the *P. guajava *var. pomifera essential oil.

Compound	RT (min)	IK	(%)
Benzaldehyde	3.93	952	0.99
1,8-Cineole	5.03	1009	13.31
Linalool	6.21	1117	0.39
*α*-Terpineol	8.37	1178	2.21
(E)-Caryophyllene	14.14	1411	9.51
(Z)-Caryophyllene	15.00	1419	1.49
Eudesmen-4-ol	15.83	1448	6.65
*α*-Guaiene	16.04	1461	5.06
Nerolidol	17.54	1556	3.49
Caryophyllene alcohol	17.95	1570	0.54
Caryophyllene oxide	1828	1580	10.21
Selina-6-en-4-ol	18.95	1588	3.05
Alloaromadendrene oxide	19.43	1646	4.05
Isoaromadendrene oxide	19.52	1648	11.13
Cadinol	19.60	1669	2.49
Epiglobulol	19.95	1688	19.20

		Total	93.77

**Table 2:** Oxidative stress markers and activity of enzymes involved in the antioxidant metabolic routes in *D. melanogaster *exposed to the *P. guajava* var. pomifera essential oil.

	TBARS	ROS	PSH	NPSH	GST	SOD	CAT
Control 3 h	1.2 ± 0.2	100 ± 12.8	5.7 ± 0.8	1.8 ± 0.2	109.6 ± 6.7	60.4 ± 3.9	42.7 ± 7.2
Oil 3 h	1.4 ± 0.2	127 ± 6.9^*^	5.9 ± 1.1	2.7 ± 0.2^*^	122.5 ± 10.7	69.3 ± 11.8	45.6 ± 6.1

Control 6 h	1.4 ± 0.1	100 ± 8.9	6.2 ± 0.9	1.9 ± 0.1	116.7 ± 12.4	56.5 ± 5.6	41.3 ± 2.8
Oil 6 h	1.5 ± 0.1	144.7 ± 10.2^*^	6.7 ± 0.8	2.1 ± 0.1	155.4 ± 14.5^*^	58.2 ± 12.6	66.8 ± 12.9^*^

Control 12 h	1.3 ± 0.1	100 ± 7.5	5.6 ± 0.3	2.1 ± 0.1	118.1 ± 7.7	58.4 ± 5.9	48.8 ± 3.2
Oil 12 h	1.9 ± 0.3^*^	167 ± 11.3^*^	5.7 ± 0.4	2.3 ± 0.1	179.7 ± 23.8^*^	77.2 ± 15.4	58.3 ± 7.6^*^

TBARS: nmol mg^−1^ protein^−1^.

ROS: percentage of control (%).

PSH and NPSH: *μ*mol mg^−1^ protein^−1^.

Enzyme activity: mU mg^−1^ protein^−1^.

^*^ *P* < 0.05 compared to control.
